# Model for *B*1 Imaging in MRI Using the Rotating RF Field

**DOI:** 10.1155/2014/461647

**Published:** 2014-05-19

**Authors:** Adnan Trakic, Jin Jin, Ewald Weber, Stuart Crozier

**Affiliations:** The School of Information Technology and Electrical Engineering, The University of Queensland (UQ), Brisbane, QLD 4072, Australia

## Abstract

Conventionally, magnetic resonance imaging (MRI) is performed by pulsing gradient coils, which invariably leads to strong acoustic noise, patient safety concerns due to induced currents, and costly power/space requirements. This modeling study investigates a new silent, gradient coil-free MR imaging method, in which a radiofrequency (RF) coil and its nonuniform field (*B*_1_^+^) are mechanically rotated about the patient. The advantage of the rotating *B*_1_^+^ field is that, for the first time, it provides a large number of degrees of freedom to aid a successful *B*_1_^+^ image encoding process. The mathematical modeling was performed using flip angle modulation as part of a finite-difference-based Bloch equation solver. Preliminary results suggest that representative MR images with intensity deviations of <5% from the original image can be obtained using rotating RF field approach. This method may open up new avenues towards anatomical and functional imaging in medicine.

## 1. Introduction

Magnetic resonance imaging (MRI) is a widely accepted tool for clinical assessment and diagnosis of various disease states due to its exceptional soft-tissue contrast and ability to image in any orientation [1]. MRI utilizes a very strong and uniform static magnetic field in which (typically hydrogen) nuclei within the patient resonate at a radiofrequency (RF) that is proportional to the static field strength. Current-driven gradient coils are then rapidly switched to spatially localize the origin of the MRI signal by a linear variation of the axial magnetic field component (and thus frequency of the MR signal) along the *x*, *y*, and *z* coordinates. Finally, transmission and reception coils operating at the same radiofrequency are placed near and around the patient to excite and receive the MR signals, respectively. The resulting signals are digitally processed using the Fourier transform to form MR images.

Gradient coil equipment is expensive/intricate, noisy, power and space demanding, and can induce complex spatiotemporal eddy currents in nearby conducting structures, including the patient [2–4]. Gradient switching rates are limited by regulatory agencies to avoid peripheral nerve stimulation [5, 6]. Many MRI researchers have sought after ways to completely avoid the use of gradient coils. Partially parallel imaging (PPI) has been proposed as complementary function to Fourier preparation by magnetic field gradients [7–11]. For instance, one such PPI technique known as sensitivity encoding (SENSE) utilizes complex RF coil sensitivity profiles to improve the image quality or accelerate the image acquisition time by substituting gradient encoding [12, 13]. PPI has advanced the field of MRI in the recent decade and some of its methodologies have verified imaging without gradient coils, albeit at a low image resolution [14, 15]. An alternative (and gradient coil free) imaging strategy based on gradients in the transmit RF field was proposed by Hoult [16]. Specifically, (linear) *B*_1_^+^ gradients in the RF magnetic field were used to encode spatial information into the MRI signal via a spatially dependent flip angle, where *B*_1_^+^ is the component of the magnetic field rotating in the same direction as the net magnetization. The *B*_1_^+^ field gradients are typically produced by a stationary RF transmit system, while MR signal acquisition is performed with a separate and dedicated receive RF body coil that typically has a uniform *B*_1_^−^ field. The rotating frame zeugmatography method was successfully applied to localized spectroscopy but had limited impact on clinical imaging [17]. The principal limitations involved slow imaging speed as only one data point is collected per excitation and very high peak RF power requirements in the case of large flip angle pulses. More current *B*_1_^+^ gradient techniques that have demonstrated great potentials include transmit array spatial encoding (TRASE) that utilizes *B*_1_^+^ phase gradients [18], high-speed rotary echo zeugmatography [19], and *B*_1_-gradient MRI using parallel transmit system [20].

Recent studies have demonstrated that mechanically rotating a single RF transceiver coil (RRFC) can emulate a large RF coil array by time-division-multiplexing (TDM) [21–23]. TDM-SENSE algorithm was developed to take advantage of the large number of RF coil sensitivity profiles generated over time and subsequently accelerate the image acquisition. The RRFC approach brings a number of hardware advantages (i.e., requires only one RF channel, averts coil-coil coupling interactions, and facilitates large-scale multinuclear imaging). In this novel research study, we demonstrate that the RRFC transmit concept can be successfully applied to *B*_1_^+^ gradient encoding using either linear or nonlinear RF gradients. The methodology includes flip angle and RF sensitivity-based amplitude and phase modulation schemes as modeled by an unconditionally stable, central finite-difference-based, nonlinear Bloch equation solver.

## 2. Materials and Methods

### 2.1. *B*_1_^+^ Encoding: Simplistic Model

In this modeling study, it is assumed that a single RF transmit coil (with a *B*_1_^+^ field gradient) is rotating about the sample at the angular frequency *ω*_*rot*⁡_, while the MR signal acquisition is performed with a dedicated receive RF body coil that has a uniform *B*_1_^−^. While in [20] each data point is measured once the entire RF pulse is played out, in this study we shall assume that the data points can be acquired as the RF coil is rotating (with an active *B*_1_^+^ field). An example of a practical implementation of this concurrent transmission-reception approach is provided at the end of the Section 4. Consequently, the aim of the present study is to investigate the feasibility of *B*_1_^+^ encoding with the rotating RF coil (RRFC), whilst the MR signal reception is assumed to be intrinsic to the problem.

With *B*_1_-RRFC a single data point can be measured per rotated *B*_1_^+^ field (gradient), thereby filling the 3-dimensional (3D) pseudo *k*-space:

(1) $$\begin{array}{c} {D_{t}}_{\;}\sim \overset{}{\underset{r}{\int }}M\left(r\right)\frac{B_{1\theta \left(t\right)}^{+}\left(r\right)}{\left|B_{1\theta \left(t\right)}^{+}\left(r\right)\right|}\sin\left(\gamma tg_{\alpha (t)}\Delta \left|B_{1\theta \left(t\right)}^{+}\left(r\right)\right|\right)dr, \end{array}$$

where *D*_*t*_ is the measurement (i.e., complex data point), *M*(**r**) is the signal intensity at the position vector **r**, *γ* is the gyromagnetic ratio, *t* denotes the time parameter, **B**_1*θ*(*t*)_^+^(**r**) is the complex RF transmit magnetic field at coil position *θ*(*t*) = *ω*_*rot*⁡_ *t*, Δ|**B**_1*θ*(*t*)_^+^(**r**)| is the unit *B*_1_^+^ transmit field at angular position *θ*, and *g*_*α*(*t*)_ is the *B*_1_^+^ field scaling factor, which is function of time and part of the encoding sequence.

Image reconstruction from *k*-space is performed via matrix inversion of (1):

(2) $$\begin{array}{c} D=EG, \end{array}$$

with *D* a vector containing *I* = *t* complex data points, *G* containing the values of magnetization *M*(**r**) discretized on a spatial grid of *J* = *NML* voxels, and *E* the *J* × *I* encoding matrix:

(3) $$\begin{array}{c} E_{j,i}=\frac{B_{1\theta \left(i\right)}^{+}\left(r_{j}\right)}{\left|B_{1\theta \left(i\right)}^{+}\left(r_{j}\right)\right|}\sin\left(\gamma tg_{\alpha (i)}\Delta \left|B_{1\theta \left(i\right)}^{+}\left(r_{j}\right)\right|\right), \end{array}$$

where (*N*, *M*, *L*) are the discrete dimensions of the MR image. The desired distribution *M*(**r**) can be reconstructed via solving (3). One way to do so is via the (regularized) pseudo-inverse denoted by +:

(4) $$\begin{array}{c} G=E^{+}D, \end{array}$$

where image *M*(**r**) is obtained by restricting the 1-dimensional vector *G* to a 3-dimensional matrix.

### 2.2. *B*_1_^+^ Encoding Using the Nonlinear Bloch Equation

In MRI, when small flip angle is employed, the RF pulse design can be easily approximated by the inverse Fourier transformation, which is a linear process. Such approximation fails at higher flip angles (typically above 30°) and the complete solution of the nonlinear Bloch equation is required. Herein, we employ an iterative, unconditionally stable, finite-difference-based numerical solution of the Bloch equation (∂**M**/∂*t* = *γ* **M** × **B**) centered around the midpoint of the discretization time step Δ*t*, as developed and described in detail in [24]:

(5) $$\begin{array}{c} M^{n+1}=M^{n}+\frac{\gamma \Delta t}{1+\left(1/4\right)\gamma^{2}\Delta t^{2}B^{2}}\left(M^{n}+\frac{1}{2}\gamma \Delta t\;M^{n}\times B\right)\times B, \end{array}$$

where $B=B_{x}\left(r,t\right)\hat{i}+B_{y}\left(r,t\right)\hat{j}+B_{z}(r,t)\hat{k}$ describes the magnetic field vector at a particular point in space and time and *B* is the magnitude of **B**. Expanding (5) including the cross product term (**M** ^*n*^ + (1/2)*γ*Δ*t* **M** ^*n*^ × **B**) × **B** will result in three Cartesian components of net magnetization (i.e., *M*_*x*_(**r**, *t*), *M*_*y*_(**r**, *t*), and *M*_*z*_(**r**, *t*)).

The eigenvalues of the matrix determine the stability of the iteration scheme. It can be shown that the scheme is unconditionally stable with eigenvalues [24]:

(6) $$\begin{array}{c} {\;\;\;\;\;\;\;\;\;\;\lambda }_{1}=1,\;\;\lambda_{2}=\lambda_{3}^{*}=\frac{1+0.5iB\gamma \Delta t}{1-0.5iB\gamma \Delta t}, \end{array}$$

with all eigenvalues having moduli equal to one.

A pseudo-inverse solution was modeled in Matlab by populating the encoding matrix *E* with rotated *B*_1_^+^ field data according to (3) and then solving the system (2) using the Matlab* pinv* function (i.e., see (4)). Instead of applying (1) and the linear map equation (3), the nonlinear Bloch-LSQR solution was modeled by populating the matrix *E* whilst calling the Bloch function written in Matlab script, followed by the solution of (2) by using the* lsqr* method in Matlab. The rotation of the complex sensitivity map over time, **B**_1*θ*(*t*)_^+^(**r**), was implemented in the Bloch equation as part of the discrete time-stepping procedure of the term **B** in (5). The results obtained with pseudo-inverse and Bloch-LSQR method were subsequently compared.

### 2.3. Experiment 1

Initial models assumed a 2-dimensional Matlab's Shepp-Logan phantom (The Mathworks) of 5 mm in-plane resolution with a matrix size of *N* × *M* = 64 × 64 pixels and a unit 37.5 nT/m linear *B*_1_^+^ field gradient within the imaging region produced by the single-channel RF coil of the RRFC transmit system rotating about the phantom at a frequency of *ω*_*rot*⁡_ = 1256 rad s^−1^. The gradient was defined under the assumption that, at the midpoint of the longest axis of the phantom, the *B*_1_^+^ amplitude is one quarter the maximum *B*_1_^+^ magnitude (i.e., |*B*_1_^+^_max⁡_|/4). The rotation of the linear sensitivity map was performed with the Matlab's imrotate.m function; while for every incremental map rotation, ±0.2% of random noise was added to simulate the noise propagation. An RF gauss pulse *b*(*t*) ≡ *b*(*θ*) modulated the *B*_1_^+^ field according to **B**_1*θ*_^+^(**r**) = *b*(*θ*)**B**_1*θ*_^+′^(**r**). The duration of the entire RF pulse was set to 5 ms and is assumed to correspond to one period of coil rotation. We assumed that only a *z*-gradient coil was used in conjunction with the RF pulse to select the axial slice of magnetization, in the absence of transverse gradient coils. The unit *B*_1_^+^ field was then incremented after each period of RF coil rotation (i.e., phase encode) in steps of *g*_*α*_ = *α* − *M*/2 − 1/2, so that at steps *α* = 1 and *α* = 64, the net magnetization would be rotated by the flip angle *φ* = −90° and *φ* = 90°, respectively. In addition, after each phase encoding (i.e., time of repetition (TR)), the RF pulse was assumed to start at a new angular coil position with an offset of Δ*θ* = 360°/*M* ≈ 5.625°, which ensured that in conjunction with the amplitude-varying RF pulse, the resulting image of the sample was uniformly weighted (“illuminated”) following the matrix inversion of (4). For instance, if the RF coil was to start to rotate for one period from the same angular position on each TR, one side of the sample would be “illuminated” more than another by the RF pulse. TR was assumed to be sufficiently long to enable complete signal recovery to thermal equilibrium. Matrices *D* and *E* were formed and matrix system (4) was solved using both Matlab's pseudo-inverse and the least-squares QR approach (*lsqr.m*), in which case the nonlinear Bloch equation solver was applied with 20 time steps and Δ*t* = 250 *μ*s. The initial values of magnetization were assumed as follows: *M*_*x*_ = 0, *M*_*y*_ = 0, and *M*_*z*_ = 1A/m. A number of solutions with unit *B*_1_^+^ gradient varying up to 10^−12^ T/m were performed to quantify the relative deviation of the resulting image from the original image. With different unit gradients, the maximum flip angle (FA) may be smaller or larger than 90°. In this case we were only interested in the reconstruction performance relative to the maximum achievable FA.

### 2.4. Experiment 2

Since spatial sensitivity profiles of RF coil systems are usually nonlinear, it is essential to perform experiments using nonlinear RF field gradients. Here, numerical modeling synonymous to experiment 1 was conducted using a realistic (magnetization-normalized) human head image (*N* × *M* = 80 × 80 pixels) with available *k*-space data (2T Whole-body MRI, The University of Queensland (UQ), Australia). A hybrid Method-of-Moments/Finite-Element Scheme [25] simulated the complex sensitivity map of a surface coil loop at 85.45 MHz (2 Tesla), as shown in Figure 4(a). The sensitivity map was rotated at *ω*_*rot*⁡_ = 90 rad s^−1^. An RF gauss pulse of 5 ms in duration was applied synonymously to Experiment 1. The experiment was repeated *M* = 80 number of times, wherein the strength of the pulse was incremented with each phase encoding step according to *g*_*α*_ = *α* − *M*/2 − 1/2. The RF pulse was adjusted to generate a maximum FA = 90°.

### 2.5. Experiment 3

**Figure 1:**
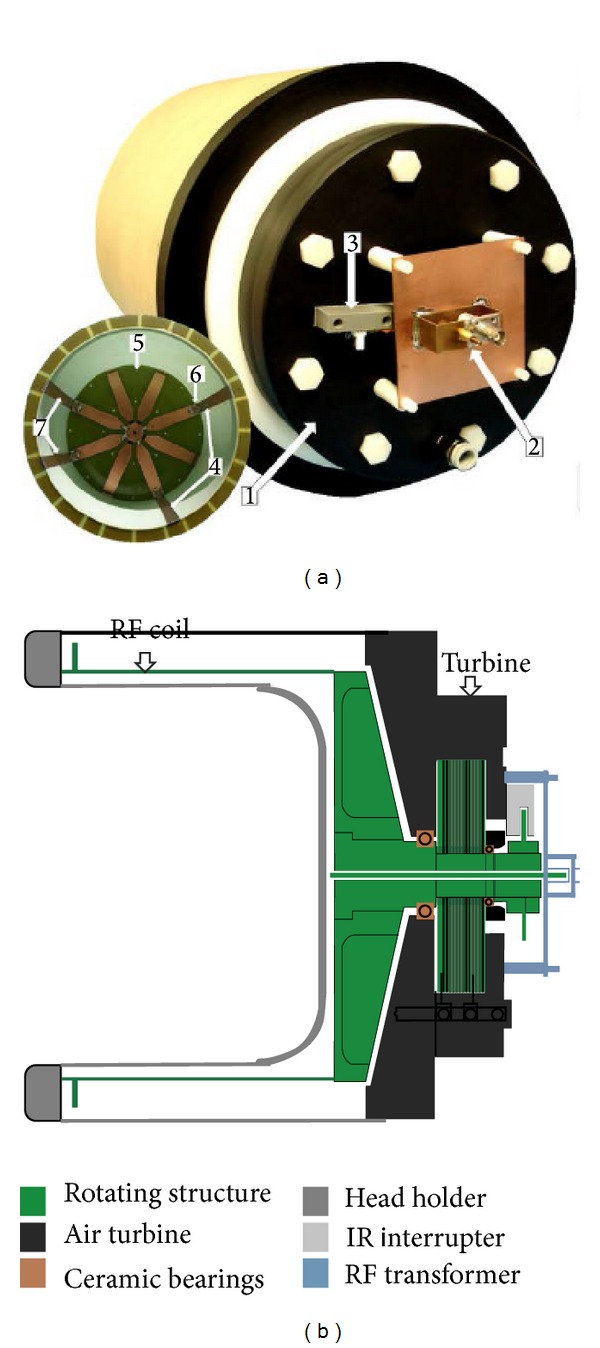
(a) Front and rear views of the actual RRFC system designed for head imaging, measuring approximately 340 mm in diameter and 480 mm in length, and showing custom-made air turbine (1), the inductively coupled RF link, (2) and infrared (IR) photo-interrupter (3). The front view of the disassembled rotating cylinder shows the RF surface coil loop (4) that is connected to a printed circuit board (5) situated at the end of the rotating cylinder and is tuned by a variable capacitor network (6). (b) Schematic diagram of RRFC in side-view showing the major system components.

To obtain realistic sensitivity maps and *k*-space data for the purpose of this study, we employed a previously constructed rotating RF coil (RRFC) apparatus for head imaging, as shown in Figure 1. The RRFC apparatus consists of a pneumatically rotated RF coil capable of RF transmit-receive operations. The engineering specifications of the MR-compatible RRFC system are beyond the scope of the current paper and have already been provided in detail in two previous publications [21, 22]. Imaging experiments were performed in a 2 Tesla whole-body MRI system (UQ, Australia) equipped with Bruker ParaVision 4.0 software. In order to perform *B*_1_^+^ encoding with RRFC it is essential to estimate the RF coil transmit sensitivity map *B*_1_^+^ (both magnitude and phase) corresponding to a large number of angular RF coil positions.

Since it is impractical to measure a large number of sensitivities, we opted to estimate at least one map and rotate it numerically to engender many other profiles. The straightforward way is to fix the RF coil at a known angular position relative to the subject, obtain the (complex-valued) MR image, and then divide the image by a uniform reference image obtained with a body coil [13]. Alternatively, an image obtained with the RF coil in receive mode can be divided by a uniform reference image to obtain the receive map *B*_1_^−^, which according to the reciprocity theorem [26], at low magnetic field strengths (<3 Tesla), is closely equivalent to the complex conjugate of *B*_1_^+^. Both methods work equally well, and in this study we used the latter approach in four independent imaging experiments by fixing the RRFC coil at angular positions interspaced by 90° and employing the fast low angle shot (FLASH) imaging sequence, parameters of which are given in Figure 7.  *B*_1_^+^ field maps were subsequently obtained after postprocessing operations involving thresholding, polynomial fitting, and phase unwrapping. To generate the pseudo *k*-space data, we rotated the sensitivity map at *ω*_*rot*⁡_ = 90 rad s^−1^ and applied a gauss RF pulse of 5 ms in duration. The experiment was repeated *M* = 100 number of times, wherein the strength of the pulse was incremented with each phase encoding step according to *g*_*α*_ = *α* − *M*/2 − 1/2. The RF pulse power was adjusted to generate a maximum FA = 90° within the field of view (FOV) of 35 × 35 cm when *α* = 100. In addition, after each phase encode (i.e., time of repetition (TR)), the RF pulse was assumed to start at a new angular coil position with an offset of Δ*θ* = 360°/*M*. The elements in matrix *E* were then populated according to (3) by rotating the *B*_1_^+^ sensitivity map via complex plane rotation and spline interpolation routines. System (2) was solved iteratively using the least-square QR factorisation method. During the computational process, the region of the anatomical head was defined by the thresholded image mask, similar to the conventional SENSE reconstruction algorithm [12]. Consequently, the resulting matrix *E* is rectangular, as it is reduced in size from the original number of columns. This method has proven to be quite effective in mitigating the noise contribution in the reconstructed image.

### 2.6. Experiment 4

To investigate the feasibility of true 3-dimensional (3D) *B*_1_-RRFC encoding in conjunction with realistic RF pulses (such as* sinc* and* sech*) in complete absence of gradient coils, we used the 3D RF field profile from Experiment 2 and defined a three-layer rectangular phantom (see Figure 10(a)) within a region of 21 × 21 × 21 voxels. The RF coil was assumed to rotate at *ω*_*rot*⁡_ = 628 rad s^−1^ and the magnitude of a 5 ms long RF* sinc* pulse was varied according to *g*_*α*_ = *α* − *M*/2 − 1/2. After each TR, the RF pulse was assumed to start at a new angular coil position with an offset of Δ*θ* = 360°/*M* ^2^. For every incremental 3D map rotation, ±0.2% of random noise was added to simulate the noise propagation. The 3D-Bloch-LSQR solver was applied with the initial magnetization conditions of *M*_*x*_ = 0, *M*_*y*_ = 0, and *M*_*z*_ = 1A/m.

## 3. Results

### 3.1. Experiment 1

**Figure 2:**
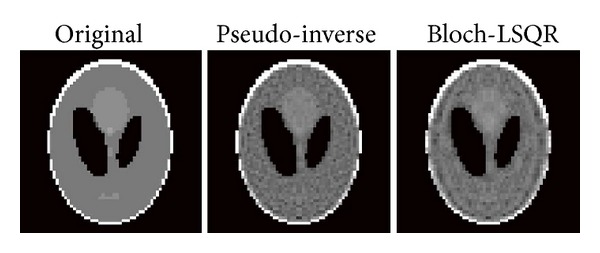
Linearly polarized *B*_1_-RRFC encoding reconstructed phantom images (*N* × *M* = 64 × 64) using the pseudo-inverse and least-square approach.

Figure 2 shows the *B*_1_-RRFC encoding results using the pseudo-inverse and the least-square (LSQR) method. The Bloch-LSQR method was stopped at 400 iterations or when the result converged to a residual of 10^−6^.

**Figure 3:**
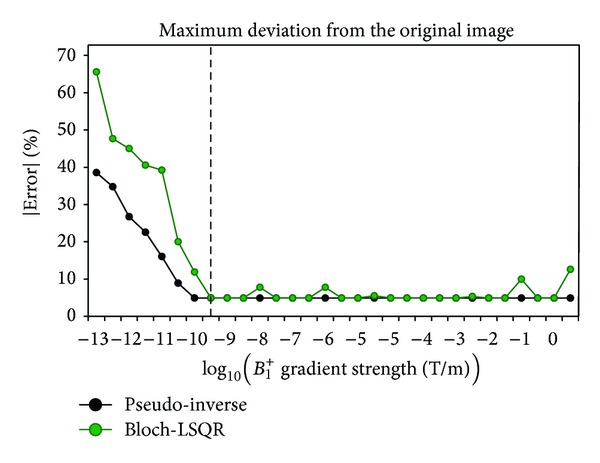
Performance evaluation of the pseudo-inverse and Bloch-LSQR method versus the strength of encoding *B*_1_^+^-gradient. The encoding system performs best (<5%) when the unit *B*_1_-gradient is at least 10^−9^ T/m.

To complete the simulation in Matlab, it took around 2.1 min/788MB RAM and 9.6 min/823MB RAM to solve (4) using the pseudo-inverse and Bloch-LSQR approach, respectively.  Figure 3 is the intensity deviation plot of the resulting image from the original image in percent versus the unit *B*_1_^+^ gradient field in T/m. We note that the deviation converges to a minimum value of 4.92% when the unit *B*_1_^+^ gradient is larger than about 1 nT/m.

### 3.2. Experiment 2

**Figure 4:**
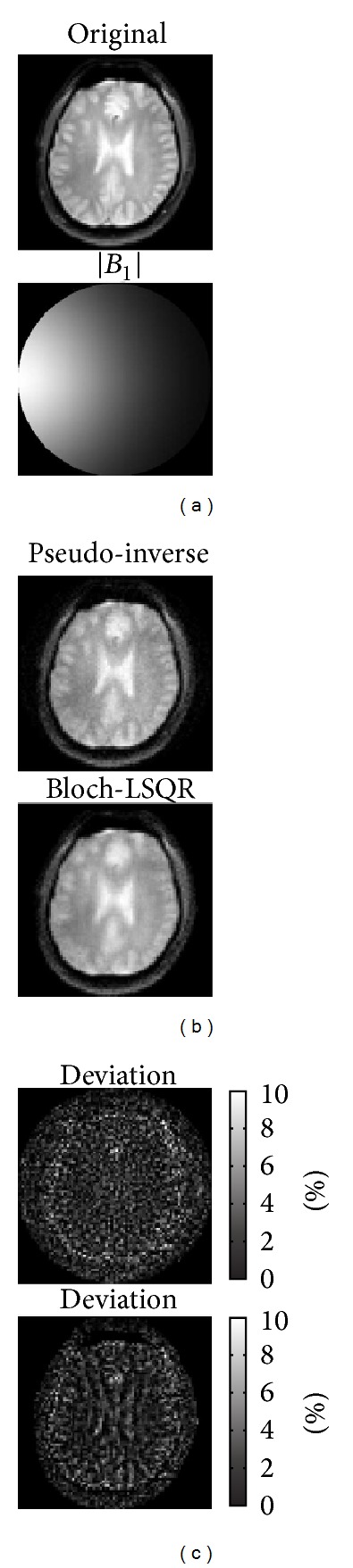
(a) Original image and simulated sensitivity map, (b) comparison of pseudo-inverse and Bloch-LSQR solved *B*_1_-RRFC image reconstruction results, and (c) the corresponding deviation maps from original image (in |%|).

Figure 4 shows the *B*_1_-RRFC image reconstruction results based on a simulated nonlinear *B*_1_^+^ field produced by a surface coil loop at 85.45 MHz. Specifically, Figure 4 compares the image reconstructions with the pseudo-inverse and Bloch-LSQR method and shows the associated percentage deviation maps of the resultant head image from the original image (obtained with sum-of-squares (SoS) method [12]). It took around 6.4 min (2.36 GB of RAM) and 10.85 min (2.44 GB of RAM) to solve (4) with the pseudo-inverse and Bloch-LSQR approach, respectively.

**Figure 5:**
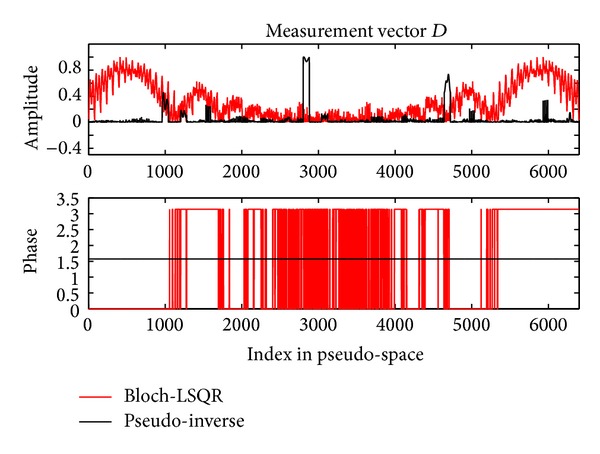
Measurement vector *D* for *N* × *M* = 80 × 80 image. Shown are both amplitude and phase values obtained with (1). The RF coil was rotated 80 times while each time a different strength *g*_*n*_ of the *B*_1_^+^-field was applied (with a total of 80 values spanning the range [*n* − *M*/2 − 1/2]).

The maximum percentage intensity deviation of the pseudo-inverse and Bloch-LSQR reconstructed images from the magnitude normalized original image was 8.2% and 8.5%, with a mean/standard deviations of −0.38/1.42 and −0.36/1.89, respectively.  Figure 5 shows the measurement vector *D* (Figure 4) both in amplitude and phase, obtained as part of the pseudo-inverse and nonlinear Bloch-LSQR solvers.

### 3.3. Experiment 3

**Figure 6:**
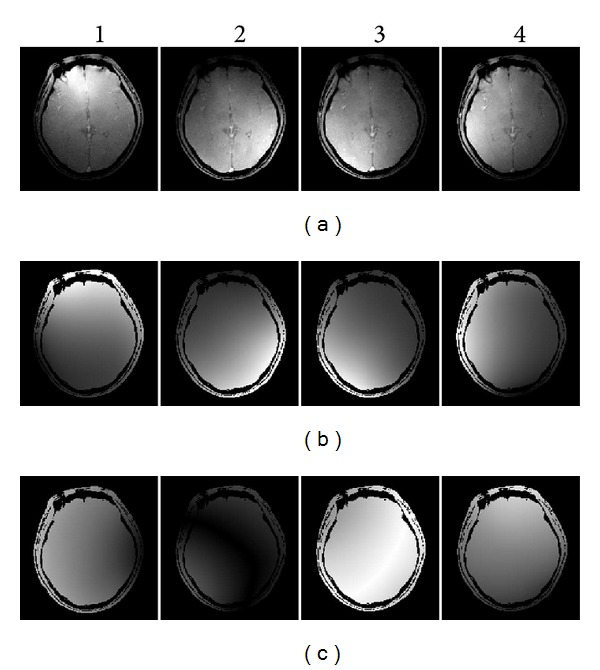
(a) Magnitude plots of raw axial head images obtained with the RF coil located at four positions with an equal angular interspacing of 90°. (b) and (c) are normalized magnitude and phase plots of the RF coil sensitivity profile obtained by dividing each raw measurement in (a) by the uniform brain reference, followed by thresholding and polynomial fitting. For each of the four images, fast low angle shot (FLASH) imaging sequence was employed with the following parameters: TR = 100 ms, TE = 8.19 ms, field of view (FOV) = 35 × 35 cm, image size (*N* × *M*) = 256 × 256, slice thickness (ST) = 5 mm, and flip angle (FA) = 30°.

Figure 6(a) shows axial head images obtained by fixing the rotating RF coil at four angular positions interspaced by 90°.

**Figure 7:**
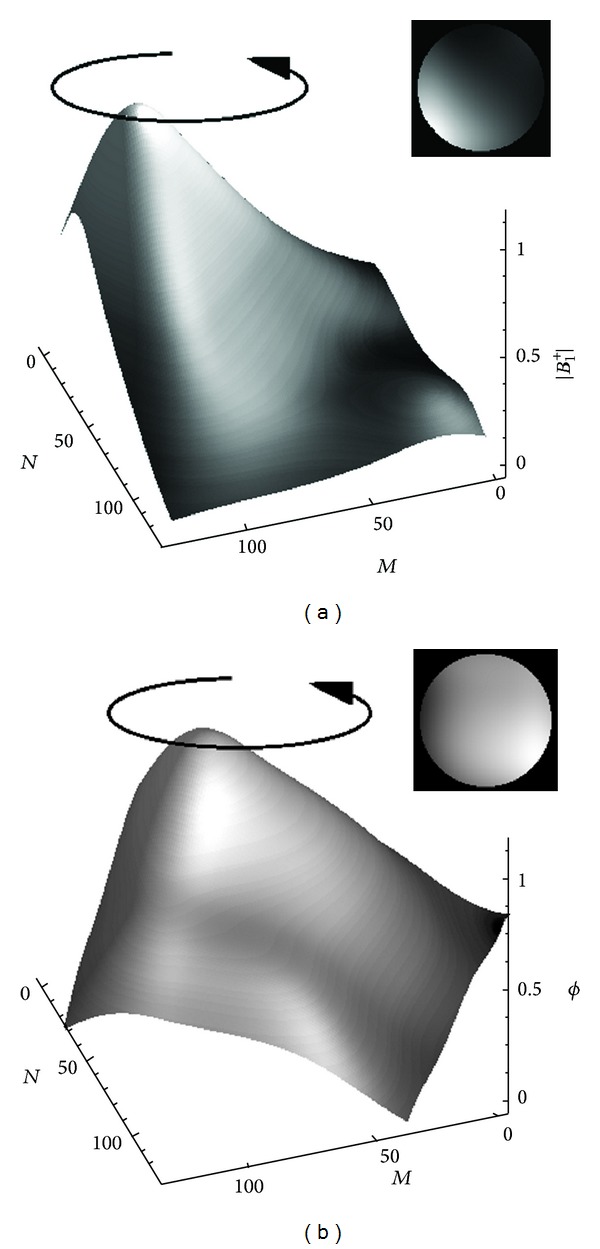
Surface plots of polynomial-fitted, nonlinear *B*_1_^+^ field profiles of normalized (a) magnitude and (b) unwrapped phase. Corresponding 2-dimensional field profiles within a circular region of interest (ROI), which is just larger than the anatomical head in Figure 6, are additionally shown to better depict the *B*_1_^+^ field behaviour in the proximity of the subject.

**Figure 8:**
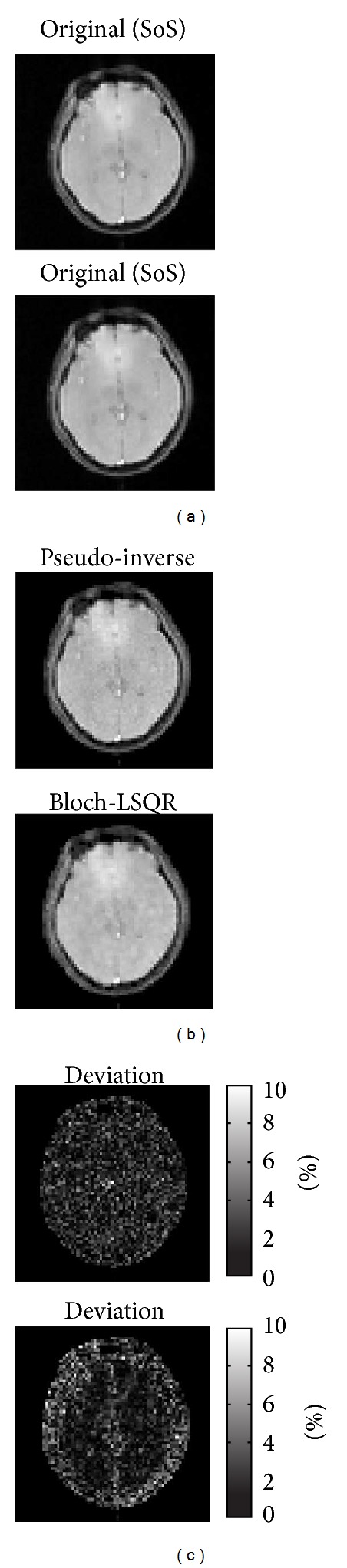
Comparison of *B*_1_-RRFC image reconstruction results including the (normalized) original, pseudo-inverse, and Bloch-LSQR as well as the absolute value intensity deviation maps (from the original image) in |%|. The results are based on the experimentally acquired sensitivity map in Figure 7. The image size is *N* × *M* = 100 × 100.

Each of the four results was divided by a uniform brain reference to obtain the raw sensitivity maps. The resulting maps were then refined by thresholding and polynomial fitting to obtain smooth magnitude and phase profiles, as illustrated in Figures 6(b) and 6(c). The magnitude and phase maps from Figure 6 were subsequently employed to estimate the *B*_1_^+^ field profile that when rotated would optimally emulate any sensitivity profile as function of RF coil angular position. The magnitude and phase profiles of the resulting spatially nonlinear *B*_1_^+^ field are shown in Figure 6. Rotating the fields of Figure 7 and incorporating angular position dependent phase (i.e., the phase relative to the *x*-*y* coordinate system of the MRI scanner) produce the four corresponding magnitude and phase profiles of Figures 6(b) and 6(c) with 96.8% accuracy.  Figure 8 shows *B*_1_-RRFC image reconstruction results based on the practical (nonlinear) *B*_1_^+^ sensitivity map (Figure 7) produced by the rotating RF coil system for head imaging. Two different image approaches to solving the system matrix equation (4) are employed, namely, the pseudo-inverse method and the Bloch-based least-squares QR factorization method (LSQR), and the image results are contrasted against an original image obtained with the sum-of-squares method [12]. To solve (4) using the pseudo-inverse (Bloch-LSQR) approach, it took around 12.5 min (21.2 min) and 4.61 GB (4.77 GB) of RAM.

Figure 8 shows *B*_1_-RRFC image reconstruction results based on the practical (nonlinear) *B*_1_^+^ sensitivity map (Figure 7) produced by the rotating RF coil system for head imaging. Two different image approaches to solving the system matrix equation (4) are employed, namely, the pseudo-inverse method and the Bloch-based least-squares QR factorization method (LSQR), and the image results are contrasted against an original image obtained with the sum-of-squares method [12]. To solve (4) using the pseudo-inverse (Bloch-LSQR), it took around 12.5 min (21.2 min) and 4.61 GB (4.77 GB) of RAM.

**Figure 9:**
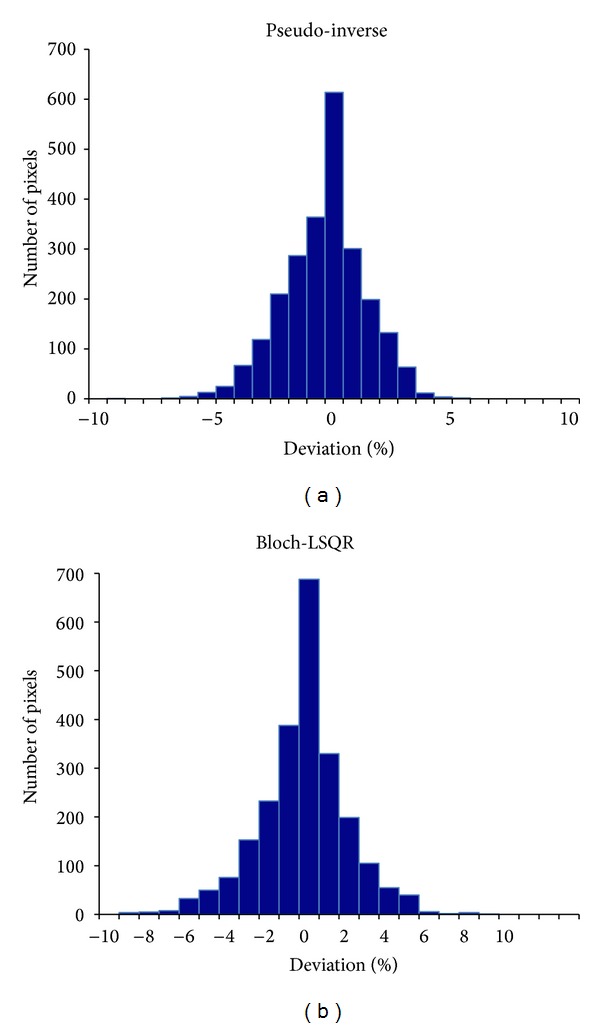
Pixel frequency distribution versus normalized percentage deviation.

The maximum percentage intensity deviations of the pseudo-inverse and Bloch-LSQR reconstructed images from the magnitude normalized original image were 9.2% and 8.7%, respectively.  Figure 9 is the frequency distribution plot of the normalized intensity deviation in % from the original image for both pseudo-inverse and Bloch-LSQR. The mean (standard deviation) values are −0.50 (1.65) and −0.56 (2.16) for pseudo-inverse and Bloch-LSQR, respectively.

### 3.4. Experiment 4

**Figure 10:**
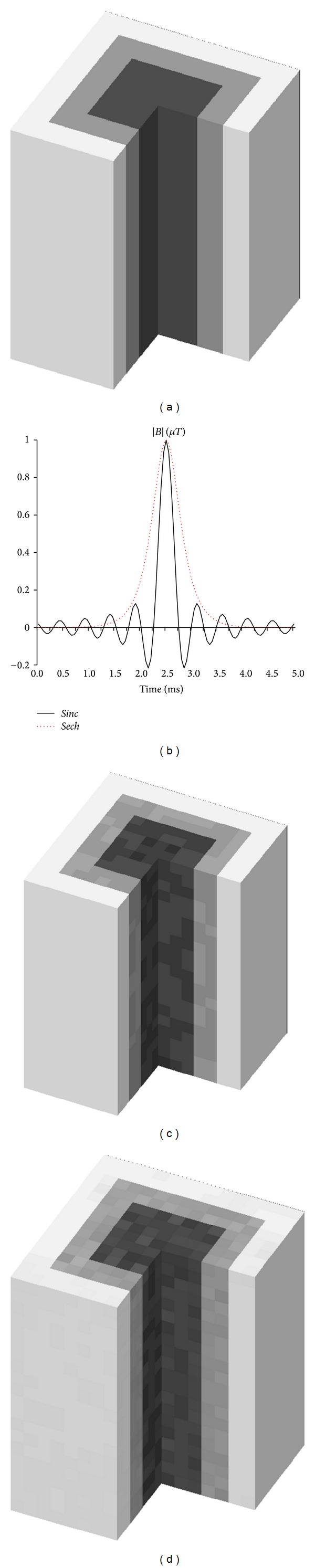
3D-*B*_1_-RRFC encoded results using Bloch-LSQR solver: (a) original 3D image, (b) normalized* sinc* (line) and* sech* (dotted line) RF pulses (absolute magnetic flux density in *μ*T over time), (c) 3D-*B*_1_-RRFC using* sinc*, and (d) 3D-*B*_1_-RRFC using* sech* RF pulse.

Figure 10 shows the results of 3D-*B*_1_-RRFC encoding using two different RF pulses (*sinc *and parabolic* sech*) and Bloch-LSQR solver, where Figure 10(a) is the cut-out of the original 3D image of the rectangular (3-layer) sample. By rotating the 3D sensitivity map of the RF coil in conjunction with the incremental amplitude adjustment of the RF pulse, we obtain the 3D-*B*_1_-RRFC encoded images of the sample.

Figures 10(c) and 10(d) are results obtained with 5 ms long* sinc* and* sech* RF pulses, respectively, after the Bloch-LQSR algorithm was applied to solve the matrix system. For a 3D image of 21 × 21 × 21 voxels in this case, 3D-*B*_1_-RRFC encoding takes approximately 2 h and 1.22 GB of RAM to complete. While for 3D *B*_1_^+^ gradient encoding simple rectangular RF pulses can be applied; shaped RF pulses such as* sinc* and* sech* were engaged primarily for exemplification purposes and to study the effect of different pulse frequency responses on the reconstructed image. In particular, compared to the rather rectangular frequency response of the* sinc* pulse (i.e., bandwith-time product Δ*fτ* ≈ 10.5), the applied* sech* pulse had a smaller bandwidth-time product (Δ*fτ* ≈ 4.2) and a stronger suppression of higher frequency components. While both filters are effective in mitigating the undesired noise in the pseudo *k*-space, compared to the* sinc* pulse, the* sech* pulse produced a 24% higher signal to noise ratio (SNR) at the expense of a slightly blurred image compared to the* sinc* pulse.

## 4. Discussion

Figures 2, 4, and 8 demonstrate that the *B*_1_-RRFC could provide representative MR images without the application of transverse *B*_0_ gradients. Since the numerical simulations take considerable time to be solved, it would be viable to accelerate the computation by a factor of up to one hundred by employing parallel computing or graphical processing units as detailed in [27, 28]. The use of the pseudo-inverse to solve (4) results in intensity deviations from the original image that are smaller than when LSQR or Bloch-LSQR approaches are applied. This is particularly true for *B*_1_^+^ gradients that are smaller than 10^−9^ T/m, as shown in Figure 3. According to Figure 3, the intensity deviation from the original image converges to about 4.92% for unit *B*_1_^+^ gradients larger than about 1 nT/m, which are easily achieved in practice. Taking the nonlinear behavior of spin magnetization into account, the images obtained with the combined Bloch-LSQR method are blurred (see Figure 4), which indicates that high-frequency components of the pseudo *k*-space are somewhat attenuated. From results in Figure 4, the pseudo-inverse solution yielded a 3.5% lower maximum percentage deviation from the original (sum-of-squares) image than the Bloch-LSQR approach.  Figure 5 further indicates the difference between the pseudo-inverse and Bloch-LSQR solution in terms of the measurement vector *D*, in that the Bloch-LSQR method takes the nonlinear behavior of the spin system into account, especially near the edges of the phantom where the flip angles are higher. From Figures 8 and 9 we note that the intensity deviations of the resulting image from the original image are modest with maximum mean and standard percentage deviation values of 0.56 and 2.16, respectively. The maximum intensity deviation was less than 9.2% in the FOV iso-centre due to the numerical error introduced by the sensitivity map rotation at the pivot point.

The method in Section 2.1 is appropriate in applications that use the small-tip angle approximation (i.e., FA < 30° or so) as the longitudinal and transverse magnetization components can be decoupled. For large flip angles, however, the complete Bloch model (Section 2.2) should be used, as it describes the complete evolution of net magnetization in space and time. The difference between the application of the simplified model (Section 2.1.) and the complete Bloch model (Section 2.2) has been illustrated in various examples of this study.

While the 2-dimensional encoding examples assumed that an ideal axial slice is already selected through the combined application of a *z*-gradient and the RF pulse, motion of the RF sensitivity profile and the complex form of the transmit RF pulse due to interleaved reception may influence the magnetization distribution in the resulting axial slice. In light of these issues, it would be therefore essential in practice to appropriately tailor the spatiotemporal RF field, the *z*-gradient, or both in order to yield a good quality slice. Figure 10 demonstrates the *B*_1_-RRFC capability of encoding 3-dimensional images of the sample without application of any *B*_0_ field gradients. 3D-*B*_1_-RRFC was accomplished by rotating the 3D sensitivity map while at the same time incrementally increasing the amplitude of a realistic RF pulse. The Bloch-LSQR solver was successfully applied in generating representative images of the 3-layer rectangular sample (see Figure 10). 3D-*B*_1_-RRFC takes a very long time to complete as the system matrix E grows as function of *N* ^3^ × *N* ^3^.

Experimentally demonstrated spatial resolution of *B*_1_^+^ gradient encoding has shown to be much higher than that provided by RF receive coil array sensitivity encoding (SENSE) alone but lower than generally achievable with *B*_0_ gradients [18]. As for any MR imaging method, increasing the sampling length, sampling rate, or the number of available degrees of freedom (DOF) as part of the reconstruction method can help increase the signal to noise ratio (SNR) and consequently improve the image quality. While rotating the RF coil (RRFC) provides a large number of DOF in form of *B*_1_^+^ gradients (RF sensitivity profiles) over time, the SNR could be further enhanced by applying a train of 180° RF refocusing pulses to refocus any decoherence in the spin magnetization. Practically, *T*_1_ and *T*_2_ relaxation phenomena and the maximum permissible specific absorption rate (SAR) will restrict the achievable number of refocusing pulses. *B*_1_^+^ gradient encoding involves both amplitude and phase modulation of the magnetization distribution. High image resolution with faithful reconstruction of small details in the final image can be achieved by sampling higher spatial frequencies. With amplitude-dominated modulation, large flip angles are required, which can generate notable SAR and corresponding temperature elevation in tissue. In order to alleviate this problem, one can increase the time of repetition (TR) or lower the fraction of amplitude modulation by enhancing the spatial phase variations in the *B*_1_^+^ gradient. Phase-dominated RF encoding is, however, not without its own limitation that is being the smooth shape of the typical phase profile. Overall, a predetermined combination of amplitude and phase modulation could provide the optimal solution regarding the compromise between the image resolution and SAR.

According to the literature [16–20], the effect of *B*_1_^+^ encoding on the image quality and resolution as function of the variation in RF amplitude and nonuniformity in RF gradient field is comparable to that of *B*_0_ gradient encoding. While *B*_1_^+^ gradient encoding has shown to be perfectly feasible at lower *B*_0_ fields (below about 1.5 Tesla), significant issues tend to arise at high static magnetic fields (3 Tesla and above), where RF fields transmitted through the patient during the image acquisition process invariably lead to significant RF field nonuniformities as result of complicated field-tissue interactions and wave phenomena [29–31]. Since at high fields *B*_1_^+^ gradient field nonuniformities are function of the angular position of the RF coil, geometry/electromagnetic tissue properties of the patient, and RF hardware setup, it becomes necessary to numerically predict and experimentally measure such nonuniformities (both in amplitude and in phase) and take the corresponding maps into account during the image reconstruction process. In fact, to achieve the desired image reconstruction, the encoding *B*_1_^+^ fields do not need to vary linearly in space, they just need to be known in advance and be sufficiently different (i.e., in this case, vary with the angular position of the RF coil). In the present study we have shown that a known and nonuniform *B*_1_^+^ field gradient can be used for purposes of successful image reconstruction.

Beside standard and spectroscopic MR imaging applications, the RRFC approach with *B*_1_^+^ gradients may be suitable for imaging sequences such as nuclear quadrupole resonance (NQR) [32], motion detection methods, echo planar rotating frame imaging (EPROFI) [19], ultrashort echo time (UTE) acquisition [33], and transmit array spatial encoding (TRASE) technique [18]. While thus far the resultant SNR of *B*_1_^+^ gradient encoded images has generally been lower than that of standard *B*_0_-gradient encoded images, with further advances in the development of *B*_1_^+^ sequences, it may be possible to improve the SNR to be of equal standard. The TRASE imaging sequence, for instance, uses multiple 180° RF refocusing pulses and phase-dominant encoding to yield a SNR that is comparable to standard MR images [18], albeit at a potentially higher SAR figure at fields higher than 0.2T at which the experiments were performed. The fundamental approach for yielding image contrast is to precondition the magnetization prior to the MR signal sampling step. In *B*_1_^+^ gradient encoding (here, with rotating RF coil), a range of contrast is available, including the typical *T*_1_/*T*_2_ relaxation times, diffusion, and chemical shift [34, 35].

While the presented *B*_1_-RRFC approach utilizes the increments in the strength of the *B*_1_^+^ gradient by increasing/decreasing the amplitude of the *B*_1_^+^ field, an alternative approach would entail the variation of the RF pulse duration *τ*, as equivalent parameter to scaling factors *g*_*α*_ and *g*_*β*_. This would be particularly useful in high field MRI where the amplitude of the RF field can lead to significant tissue heating due to high-frequency RF field-tissue interactions and dielectric resonance phenomena. Disadvantages of the *τ*-variation approach may include increased noise, off-resonance effects, and susceptibility artifact as a consequence of longer RF pulse lengths. Future studies will assess and quantify the performance of the *B*_1_-RRFC approach experimentally.

While in [20] each data point was measured once the entire RF pulse is played out, in the present theoretical study (Section 2.1) we assumed that the data points are measured during the RF pulse, akin to the continuous MR signal reception during the application of a readout magnetic field gradient. In practice, concurrent transmission and reception could, for instance, be accomplished by transmitting a series of narrow (rectangular) RF bursts and measuring the MR signal in between the bursts, that is, during the time when the RF transmitter chain is switched off or blanked. This particular mode of transmission and reception has been successfully implemented in the SWIFT imaging sequence [36].

## 5. Conclusions

A new *B*_0_ gradient-free MRI technique based on *B*_1_^+^ gradient encoding using the rotation of a RF coil (*B*_1_-RRFC) was described. The rotation of the RF coil provides a significant number of spatial (nonlinear) *B*_1_ gradients over time which can facilitate complex (amplitude and phase) modulation of axial net magnetization. An unconditionally stable finite-difference-based Bloch equation solver was applied to simulate the nonlinear dynamics of magnetization precession under the influence of the *B*_1_^+^ field. The results obtained suggest that representative MR images can be obtained using the *B*_1_-RRFC concept. Potential applications of this concept include silent, low-cost, and simplified (gradient coil-free) MRI equipment.

## References

[1] Callaghan PT (2006). *Principles of NRM Microscopy*.

[2] Boesch C, Gruetter R, Martin E (1991). Temporal and spatial analysis of fields generated by eddy currents in superconducting magnets: optimization of corrections and quantitative characterization of magnet/gradient systems. *Magnetic Resonance in Medicine*.

[3] Edelstein WA, Kidane TK, Taracila V (2005). Active-passive gradient shielding for MRI acoustic noise reduction. *Magnetic Resonance in Medicine*.

[4] Trakic A, Wang H, Liu F, Lopez HS, Weber E, Crozier S (2008). Minimizing the induced fields in MRI occupational workers by lowering the imager. *Concepts in Magnetic Resonance Part B: Magnetic Resonance Engineering*.

[5] International Commission on Non-Ionizing Radiation Protection (ICNIRP) (1998). Guidelines for limiting exposure to time varying electric, magnetic and electromagnetic fields (up to 300 GHz). *Health Physics*.

[6] The Institute of Electrical and Electronics Engineers (IEEE) (2002). *C95.6: Standard for Safety Levels with Respect to Human Exposure to Electromagnetic Fields (0-3 KHz)*.

[7] Wright SM, McDougall MP (2009). Single echo acquisition MRI using RF encoding. *NMR in Biomedicine*.

[8] Zhu Y, Hardy CJ, Sodickson DK (2004). Highly parallel volumetric imaging with a 32-element RF coil array. *Magnetic Resonance in Medicine*.

[9] Roemer PB, Edelstein WA, Hayes CE, Souza SP, Mueller OM (1990). The NMR phased array. *Magnetic Resonance in Medicine*.

[10] Griswold MA, Jakob PM, Heidemann RM (2002). Generalized autocalibrating partially parallel acquisitions (GRAPPA). *Magnetic Resonance in Medicine*.

[11] Sodickson DK, Manning WJ (1997). Simultaneous acquisition of spatial harmonics (SMASH): ultra-fast imaging with radiofrequency coil arrays. *Magnetic Resonance in Medicine*.

[12] Pruessmann KP, Weiger M, Scheidegger MB, Boesiger P (1999). SENSE: sensitivity encoding for fast MRI. *Magnetic Resonance in Medicine*.

[13] Katscher U, Börnert P, Leussler C, Van den Brink JS (2003). Transmit SENSE. *Magnetic Resonance in Medicine*.

[14] Lin F-H, Witzel T, Mandeville JB (2008). Event-related single-shot volumetric functional magnetic resonance inverse imaging of visual processing. *NeuroImage*.

[15] McDougall MP, Wright SM (2005). 64-Channel array coil for single echo acquisition magnetic resonance imaging. *Magnetic Resonance in Medicine*.

[16] Hoult DI (1979). Rotating frame zeugmatography. *Journal of Magnetic Resonance*.

[17] Blackledge MJ, Styles P, Radda GK (1988). The elimination of transmitter-receiver phase-twist artifacts in the phase-modulated rotating-frame imaging experiment. *Journal of Magnetic Resonance*.

[18] Sharp JC, King SB (2010). MRI using radiofrequency magnetic field phase gradients. *Magnetic Resonance in Medicine*.

[19] Casanova F, Robert H, Perlo J, Pusiol D (2003). Echo-planar rotating-frame imaging. *Journal of Magnetic Resonance*.

[20] Katscher U, Lisinski J, Boernert P, Graesslin I B1-gradient based MRI using a multi-element transmit system.

[21] Trakic A, Wang H, Weber E (2009). Image reconstructions with the rotating RF coil. *Journal of Magnetic Resonance*.

[22] Trakic A, Weber E, Li BK (2012). Electromechanical design and construction of a rotating radio-frequency coil system for applications in magnetic resonance. *IEEE Transactions on Biomedical Engineering*.

[23] Trakic A, Li BK, Weber E, Wang H, Wilson S, Crozier S (2009). A rapidly rotating RF coil for MRI. *Concepts in Magnetic Resonance Part B: Magnetic Resonance Engineering*.

[24] Olko MA, Turski ŁA (1991). Cartesian coordinates solver for Bloch-like equations. Application to symplectic and metriplectic dynamics of 1d magnetic chain. *Physica A*.

[25] Li BK, Weber E, Liu F, Crozier S Hybrid MoM/FEM method for use in MRI.

[26] Hoult DI (2000). The principle of reciprocity in signal strength calculations—a mathematical guide. *Concepts in Magnetic Resonance*.

[27] Chi J, Liu F, Weber E GPU accelerated FDTD solver and its application in B1-shimming.

[28] Wang H, Trakic A, Liu F, Li BK, Weber E, Crozier S (2008). Parallel solvers for finite-difference modeling of large-scale, high-resolution electromagnetic problems in MRI. *International Journal of Antennas and Propagation*.

[29] Mao W, Smith MB, Collins CM (2006). Exploring the limits of RF shimming for high-field MRI of the human head. *Magnetic Resonance in Medicine*.

[30] Ibrahim TS, Mitchell C, Abraham R, Schmalbrock P (2007). In-depth study of the electromagnetics of ultrahigh-field MRI. *NMR in Biomedicine*.

[31] Liu F, Crozier S (2004). Electromagnetic fields inside a lossy, multilayered spherical head phantom excited by MRI coils: models and methods. *Physics in Medicine and Biology*.

[32] Casanova F, Robert H, Pusiol D (1999). Phase-modulated rotating-frame NQR techniques for spatial encoding. *Journal of Magnetic Resonance*.

[33] Tyler DJ, Robson MD, Henkelman RM, Young IR, Bydder GM (2007). Magnetic resonance imaging with ultrashort TE (UTE) PULSE sequences: technical considerations. *Journal of Magnetic Resonance Imaging*.

[34] Solomon I (1959). Rotary spin echoes. *Physical Review Letters*.

[35] Humbert F (2001). Potentials of radio-frequency field gradient NMR microscopy in environmental science. *Journal of Industrial Microbiology and Biotechnology*.

[36] Idiyatullin D, Corum C, Park J-Y, Garwood M (2006). Fast and quiet MRI using a swept radiofrequency. *Journal of Magnetic Resonance*.

